# Nicotinamide Phosphoribosyltransferase/Visfatin Does Not Catalyze Nicotinamide Mononucleotide Formation in Blood Plasma

**DOI:** 10.1371/journal.pone.0022781

**Published:** 2011-08-03

**Authors:** Nobumasa Hara, Kazuo Yamada, Tomoko Shibata, Harumi Osago, Mikako Tsuchiya

**Affiliations:** 1 Department of Biochemistry, Shimane University Faculty of Medicine, Izumo, Shimane, Japan; 2 Center for Integrated Research in Science, Shimane University Faculty of Medicine, Izumo, Shimane, Japan; Governmental Technical Research Centre of Finland, Finland

## Abstract

Nicotinamide (Nam) phosphoribosyltransferase (NAMPT) is the rate-limiting enzyme in mammalian NAD synthesis, catalyzing nicotinamide mononucleotide (NMN) formation from Nam and 5-phosphoribosyl 1-pyrophosphate (PRPP). NAMPT has also been described as an adipocytokine visfatin with a variety of actions, although physiological significance of this protein remains unclear. It has been proposed that possible actions of visfatin are mediated through the extracellular formation of NMN. However, we did not detect NMN in mouse blood plasma, even with a highly specific and sensitive liquid chromatography/tandem mass spectrometry. Furthermore, there is no or little ATP, the activator of NAMPT, in extracellular spaces. We thus questioned whether visfatin catalyzes the *in situ* formation of NMN under such extracellular milieus. To address this question, we here determined *K_m_* values for the substrates Nam and PRPP in the NAMPT reaction without or with ATP using a recombinant human enzyme and found that 1 mM ATP dramatically decreases *K_m_* values for the substrates, in particular PRPP to its intracellular concentration. Consistent with the kinetic data, only when ATP is present at millimolar levels, NAMPT efficiently catalyzed the NMN formation at the intracellular concentrations of the substrates. Much lower concentrations of Nam and almost the absence of PRPP and ATP in the blood plasma suggest that NAMPT should not efficiently catalyze its reaction under the extracellular milieu. Indeed, NAMPT did not form NMN in the blood plasma. From these kinetic analyses of the enzyme and quantitative determination of its substrates, activator, and product, we conclude that visfatin does not participate in NMN formation under the extracellular milieus. Together with the absence of NMN in the blood plasma, our conclusion does not support the concept of “NAMPT-mediated systemic NAD biosynthesis.” Our study would advance current understanding of visfatin physiology.

## Introduction

**Figure 1:**
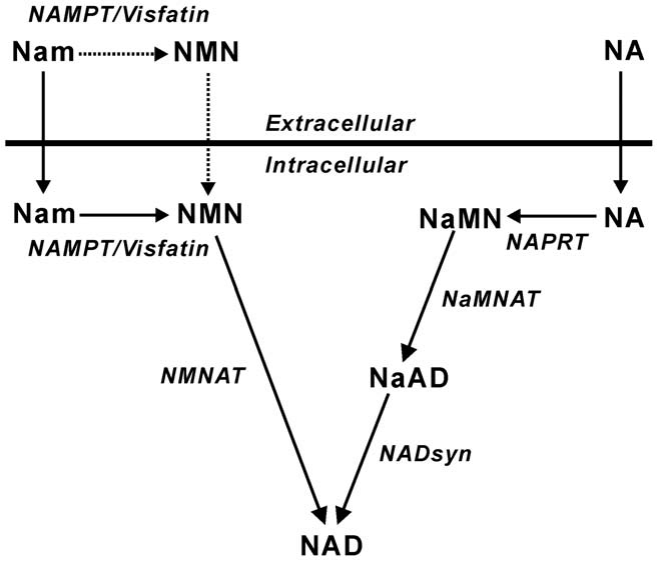
Metabolic pathways of the salvage NAD synthesis. NaAD, NA adenine dinucleotide; *NMNAT*, NMN adenylyltransferase; *NaMNAT*, NaMN adenylyltransferase; *NADsyn*, NAD synthetase. Broken arrows indicate the possible extracellular pathway in NAD biosynthesis [14].

In mammalian cells, NAD is synthesized through the *de novo* pathway from tryptophan or salvage pathways from nicotinamide (Nam) and nicotinic acid (NA) [1]. The salvage synthesis is initiated by the addition of a phosphoribosyl moiety from 5-phosphoribosyl 1-pyrophosphate (PRPP) to the respective precursors yielding the corresponding Nam and NA mononucleotides (NMN and NaMN) in the reactions catalyzed by the rate-limiting enzymes Nam phosphoribosyltransferase (NAMPT) and NA phosphoribosyltransferase (NAPRT), respectively (Fig. 1), using ATP as the activator [2]–[6].

In addition to being an intracellular NAD synthetic enzyme, NAMPT, originally identified as pre-B cell colony enhancing factor [7], has also been described as a novel adipocytokine visfatin [8], with a potential link to obesity, type 2 diabetes, and inflammatory diseases [8]–[11]. However, because of markedly conflicting associations between visfatin and these diseases in subsequent clinical studies [12], [13], the physiological significance of this protein remains largely unclear.

Revollo *et al.* have proposed that extracellular visfatin acts through “NAMPT-mediated systemic NAD biosynthesis” [9], [14]. In this proposal, it is hypothesized that Nam in blood circulation is converted to NMN by visfatin, and the resulting NMN is distributed to tissues, transported to the inside of cells, and converted to NAD to elicit reported biological effects. Subsequent studies have also suggested possible extracellular conversion of Nam to NMN by visfatin and novel functions of the resultant NMN [11], [15]. However, extracellular spaces are thought to contain little or no ATP [16]. We thus questioned whether visfatin is enzymatically active under such extracellular milieus. Indeed, the *in situ* formation of NMN by visfatin under the extracellular environments has not been directly investigated [9], [11], although enzyme activity of the secreted NAMPT was demonstrated under an *in vitro* condition with a sufficiently high concentration of ATP [9].

To address our question, we first determined *K_m_* values for the substrates Nam and PRPP in the NAMPT reaction using a recombinant human enzyme in the absence or presence of ATP. We then evaluated NAMPT activity under the intra- and extracellular milieus, based on kinetic parameters as well as the actual intra- and extracellular concentrations of the substrates and ATP we obtained in this study. We finally determined directly whether NAMPT catalyzes the *in situ* formation of NMN in mouse blood plasma. Our findings reveal that visfatin does not efficiently catalyze its reaction under the extracellular milieus.

## Materials and Methods

### Materials

[*Carbonyl*-^14^C]Nam (50 mCi/mmol) and [*carboxyl*-^14^C]NA (50 mCi/mmol) were purchased from American Radiolabeled Chemical Inc. (St. Louis, MO, USA). ATP, PRPP, and firefly luciferase and luciferin were purchased from Oriental Yeast (Tokyo, Japan), Sigma (St. Louis, MO, USA), and Wako Pure Chemical Industries (Osaka, Japan), respectively.

### Expression of wild-type human NAMPT and wild-type and mutant human NAPRT

Recombinant NAMPT and NAPRT were obtained as described previously [17]. The vector used to express mutant NAPRT, in which His-213 is replaced by Asn, was produced using a pET22b plasmid vector carrying wild-type NAPRT [17] and primers 5′-C GGG ACC CTG GCC *aac* TCC TTC GTC AC-3′ and 5′-GT GAC GAAGGA *gtt* GGC CAG GGT CCC G-3′, where the altered codon is indicated by lower-case italics. The mutant enzyme was expressed and purified as described previously [17].

### NAMPT and NAPRT assays

Enzyme preparations were incubated with PRPP and [^14^C]Nam (50 mCi/mmol) or [^14^C]NA (50 mCi/mmol) with a standard reaction mixture (30 µl) containing 50 mM Tris-Cl^−^ (pH 7.5), 10 mM MgCl_2_, 2.5 mM dithiothreitol (DTT), and 1.5 µg bovine serum albumin (BSA) in the presence or absence of specific concentrations of ATP. After incubating at 37°C for a given time, the reaction was boiled for 60 sec, and NMN and NaMN formed were separated by thin layer chromatography and quantified as described previously [17]. For kinetic analyses, enzymes were incubated with specific concentrations of PRPP together with Nam or NA in a standard reaction mixture with or without ATP at 37°C. The initial velocity was plotted against the concentration of the substrate. The curves were fitted using the non-linear regression method (DeltaGraph 5), from which the *K_m_* and *V_max_* for each reaction were derived.

### Preparation of mouse blood plasma

The protocol of blood plasma preparation was approved by the ethical committee for animal experiments of Shimane University (Permit Numbers: IZ22–43, IZ23–38), in accordance with the Guidelines for Animal Experimentation of the Japanese Association for Laboratory Animal Science. All efforts were made to minimize suffering of the animals. Blood from 6–13 week-old C57/BL mice was drawn into heparinized syringes under sodium pentobarbital anesthesia. Unless otherwise stated, the blood was immediately mixed with 1/50 volume of 125 mM EDTA/EGTA. After centrifugation at 1,500× *g* at 4°C for 7 min, the supernatant was collected and used as freshly prepared plasma.

### Determination of plasma concentrations of Nam, PRPP, ATP, and NMN

Plasma concentrations of Nam and NMN were determined by liquid chromatography/tandem mass spectrometry (LC/MS/MS) analysis. The fresh plasma was immediately mixed with an equal volume of 0.5 N perchloric acid (PCA). After centrifuging at 15,000× *g* for 5 min, supernatant was neutralized by adding an equal volume of 1 M ammonium formate (pH 6.4). The amounts of Nam and NMN in the supernatant were determined, using a triple quadrupole mass spectrometer (API3000, Applied Biosystems, Foster City, CA, USA), as described previously [18].

Plasma PRPP levels were determined by NAPRT assay as follows. The fresh plasma was immediately boiled for 60 sec, cooled on ice, and centrifuged. The supernatant (5–10 µl) was incubated with 20 µM [^14^C]NA (50 mCi/mmol), recombinant NAPRT (0.5 µg), 50 mM Tris-Cl^−^ (pH 7.5), 10 mM MgCl_2_, 2.5 mM DTT, 1 mM ATP, and 1.5 µg BSA in a final volume of 30 µl. After incubating at 37°C for 15 min, the reaction was boiled for 60 sec and the amount of NaMN formed was determined using thin layer chromatography assay, as described above. A linear relationship was observed between the amount of PRPP added to the plasma (0.05–5 µM) and that of NaMN formed.

Plasma ATP levels were determined by luciferin/luciferase assay. The fresh plasma was immediately mixed with an equal volume of 0.5 N PCA. After centrifuging, supernatant was neutralized by ammonium formate and diluted 20-fold with H_2_O. The luciferin/luciferase mixture (430 µM luciferin, 8.6 µg/ml luciferase, 22 mM Tris-Cl^−^ (pH 7.8), 8.6 mM MgSO_4_, 0.43 mM EDTA, 4.3 mM DTT, 86 µM sodium pyrophosphate, and 2.2 mg/ml BSA) was added to samples, and the sample luminescence was compared with an ATP standard curve.

### Determination of degrading activity against PRPP, ATP, and NMN in the plasma

Fresh plasma prepared without the addition of EDTA/EGTA was incubated with PRPP, ATP, or NMN in the presence or absence of 2.5 mM EDTA/EGTA. After incubating at 4 or 37°C for the indicated times, the amounts of these phosphated compounds remaining in the plasma were determined, as described above.

### *In situ* NAMPT reaction in the plasma

Fresh plasma (30 µl) prepared without the addition of EDTA/EGTA was incubated with recombinant NAMPT, PRPP, and ATP in a reaction mixture (40 µl) containing 50 mM Tris-Cl^−^ (pH 7.5) at 37°C for 10 min. After the reaction was terminated by adding an equal volume of PCA, the NMN that formed was quantified, as described above.

### Statistical analysis

Statistical data are expressed as the mean ± S.D. of *n* experiments.

## Results

### ATP is an essential activator of NAMPT reaction

**Figure 2:**
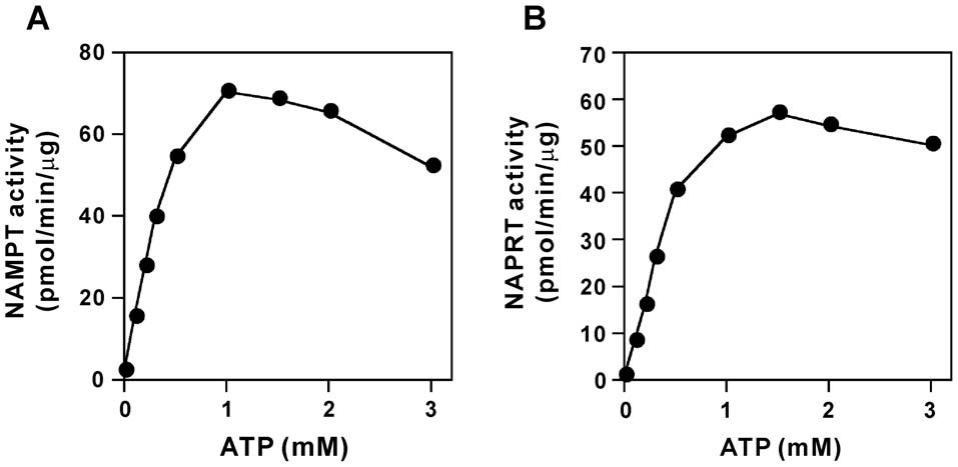
ATP is an essential activator of NAMPT and NAPRT reactions. NAMPT (A, 15 ng) and NAPRT (B, 20 ng) were incubated with 20 µM [^14^C]Nam and [^14^C]NA for 6 and 7 min, respectively, in the presence of 1 µM PRPP and indicated concentrations of ATP. The amounts of NMN (A) and NaMN (B) formed were determined. Data in figures 2 and 3 are representative of at least three experiments.

We first determined *K_m_* values for the substrates Nam and PRPP in the NAMPT reaction in the absence or presence of ATP. As shown in Table 1, *K_m_* values for Nam and PRPP dramatically decreased from 8.06 to 1.22 µM and from 23.2 to 0.51 µM, respectively, in the presence of 1 mM ATP. Taken together with the intracellular concentrations of Nam and PRPP (around 10 and 1 µM, respectively [19]–[21]), the kinetic data predicted that NAMPT would exhibit significant activity only in the presence of ATP under the intracellular condition. Indeed, when NAMPT was incubated with 20 µM Nam and 1 µM PRPP in the presence of 1 mM ATP, NMN formation was readily observed, whereas the enzyme did not exhibit significant activity without ATP (Table 2). In contrast, at much higher concentrations of Nam and PRPP (50 µM), the enzyme exhibited significant activity even in the absence of ATP (Table 2). These observations indicate that ATP is an essential activator for NAMPT at the intracellular concentrations of the substrates. As shown in Fig. 2, the millimolar levels of ATP were required for significant activity of NAMPT under the intracellular conditions. ADP, AMP, or NAD, each 1 mM in concentration, did not exhibit the stimulatory effect on NAMPT enzyme activity (data not shown). The similar requirement of ATP was observed for human NAPRT, the NAD biosynthetic enzyme also using PRPP as a substrate and ATP as an activator [4]–[6] (Tables 1 and 2, and Fig. 2). Replacing His-213 of NAPRT [17], which corresponds to His-219 in *Salmonella typhimurium* NAPRT that is the key amino acid responsible for activation by ATP [22], with Asn rendered the enzyme ATP-insensitive (Table 1).

**Table 1:** Kinetic parameters in the NAMPT and NAPRT reactions.

Enzyme	Substrate	*K_m_ (µM)*		*V_max_ (pmol/min/µg)*	
		−ATP	+ATP	−ATP	+ATP
NAMPT	Nam	8.06±0.09	1.22±0.21	39.3±17.4	39.7±14.1
	PRPP	23.2±4.78	0.51±0.16	25.9±5.1	74.1±21.1
NAPRT	NA	131±15.1	19.6±4.5	63.9±11.3	24.6±6.6
	PRPP	88.0±42.0	0.37±0.14	41.1±20.5	21.6±6.1
H213N-NAPRT	NA	441±28	430±69	57.2±7.1	40.8±8.8
	PRPP	592±165	640±189	68.5±8.4	54.7±9.1

NAMPT (36 ng) was incubated with various Nam concentrations at a fixed PRPP concentration (300 µM) for 7 and 20 min in the presence or absence of 1 mM ATP, respectively. The enzyme (7 and 36 ng) was also incubated with various concentrations of PRPP at fixed Nam concentrations (5 and 40 µM) for 7 and 30 min in the presence or absence of 1 mM ATP, respectively. NAPRT (70 and 100 ng) was incubated with various NA concentrations at a fixed PRPP concentration (300 µM) in the presence or absence of 1 mM ATP, respectively, for 30 min. The enzyme (12 and 90 ng) was also incubated with various concentrations of PRPP at fixed NA concentrations (50 and 380 µM) for 15 and 30 min in the presence or absence of 1 mM ATP, respectively. The mutant NAPRT (H213N-NAPRT, 140 ng) was incubated with various NA concentrations at a fixed PRPP concentration (600 µM) or with various concentrations of PRPP at a fixed NA concentration (650 µM) in the presence or absence of 1 mM ATP for 60 min. *K_m_* and *V_max_* values represent the mean ± S.D. of at least three separate experiments.

**Table 2:** NAMPT and NAPRT activities in the absence or presence of ATP.

Substrate concentration	Enzyme	Activity *(pmol/min/µg)*	
		−ATP	+ATP
Low	NAMPT	2.13	70.4
	NAPRT	0.87	52.0
High	NAMPT	25.9	48.1
	NAPRT	9.67	19.3

NAMPT (15 ng) and NAPRT (20 ng) were incubated with 20 µM [^14^C]Nam and [^14^C]NA for 30 min, respectively, in the presence of 1 µM PRPP (low substrate concentration) without or with 1 mM ATP. NAMPT (96 ng) and NAPRT (100 ng) were also incubated with 50 µM [^14^C]Nam and [^14^C]NA for 7 and 6 min, respectively, in the presence of 50 µM PRPP (high substrate concentration) without or with 1 mM ATP.

### In mouse blood plasma, although Nam is present, PRPP and ATP are almost absent

We next investigated whether NAMPT exhibits *in situ* enzymatic activity in an extracellular environment, blood plasma, which has been reported to contain Nam [23] but little or no ATP [16]. We determined concentrations of Nam, PRPP, and ATP in freshly prepared mouse blood plasma and evaluated NAMPT activity in the extracellular milieu, based on the kinetic parameters determined above. Using LC/MS/MS analysis [18] and the luciferin/luciferase method, we determined Nam and ATP concentrations as 4.6±1.5 µM (*n* = 3) and extremely low (1.3±0.3 µM, *n* = 3) in the plasma, respectively, which are consistent with those previously reported in mammalian circulation in general [16], [23]. With NAPRT assay, plasma concentration of PRPP was determined as below the limit of detection (<50 nM, *n* = 3). In marked contrast with the previous report [9], NMN was not detected in the plasma (<50 nM, *n* = 5) by LC/MS/MS analysis.

**Figure 3:**
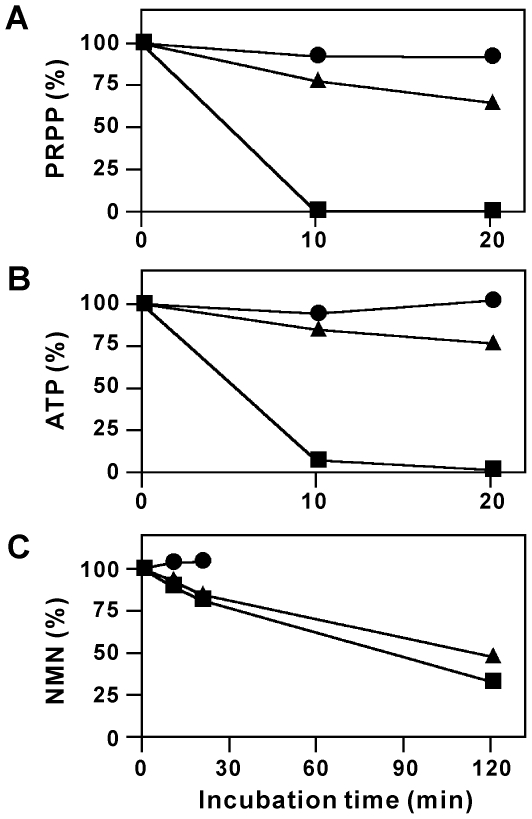
PRPP, ATP, and NMN are degraded in mouse blood plasma. PRPP, ATP, or NMN (150, 300, or 60 pmol, respectively) was incubated with the blood plasma (30 µl) in the presence of EDTA/EGTA at 4 (*circles*) or 37°C (*triangles*) or in the absence of the chelators at 37°C (*squares*) for the indicated times. After the incubation, the amounts of PRPP (A), ATP (B), and NMN (C) remaining at each time point in the plasma were determined and expressed as percent of those at 0 min.

It is well known that enzyme activities degrading phosphated compounds, including ATP, are present in blood [16]. We might not detect PRPP, ATP, and NMN in blood plasma because they were rapidly degraded by the enzymes during the plasma preparation. To test this possibility, we examined the stability of PRPP, ATP, and NMN in the plasma. As shown in Fig. 3, these compounds were not significantly degraded in the plasma for at least 20 min in the presence of EDTA/EGTA at 4°C, the conditions used to prepare plasma. After incubating even at 37°C, they were largely protected from degradation (Fig. 3). In marked contrast, PRPP and ATP were rapidly degraded in the plasma without the addition of the chelators (Fig. 3). NMN was also degraded in the absence of the chelators at 37°C, but more slowly than PRPP or ATP (Fig. 3). These results indicate that although PRPP, ATP, and NMN are degraded in the plasma, these compounds are stable if plasma is maintained at 4°C in the presence of the chelators, and thus the inability to detect these compounds in the plasma is not due to their degradation during the plasma preparation.

From all these observations, we conclude that in the mouse blood plasma, although Nam is present, PRPP and ATP are almost absent. Taken together with much lower concentrations of Nam and PRPP in the plasma compared with their intracellular levels and the low affinity of NAMPT for the substrates in the absence of ATP, NAMPT should not efficiently catalyze its reaction in the plasma.

### NAMPT does not form NMN in the blood plasma

To directly examine above notion, we incubated NAMPT with the plasma and the formation of NMN was determined. Even when NAMPT was incubated with the plasma at 25 µg/ml, a concentration 10 thousand times higher than those in blood plasma (1–3 ng/ml) [24], [25], NMN formation was not observed (*n* = 3). However, upon further addition of PRPP (30 µM) together with ATP (1 mM), a significant amount of NMN was synthesized in the reaction (0.42±0.07 µM, *n* = 3). When only PRPP (30 µM) was added to the plasma together with NAMPT, NMN formation was not observed (*n* = 3), confirming the essential requirement of ATP for the NAMPT reaction. Under the condition, PRPP remained in the plasma at 3.2±0.6 µM (*n* = 3). These observations indicate that NAMPT does not catalyze the NAMPT reaction in the plasma without the addition of both PRPP and ATP.

## Discussion

NAMPT is the rate-limiting enzyme catalyzing the first step of NAD salvage synthesis in mammalian cells [1]. Also known as visfatin, circulating NAMPT has been reported to exert a variety of actions [8]–[11], [15]. It has been proposed that possible extracellular actions of visfatin are mediated through its NAMPT activity [9], [11], [14], [15]. However, extracellular spaces are reported to contain little or no ATP [16]. We thus questioned whether visfatin indeed catalyzes NMN formation under such extracellular milieus. To address this question, it is crucially important to evaluate the requirement of ATP for NAMPT under extra- as well as intracellular conditions. Using purified recombinant human NAMPT, we here demonstrated that ATP is an essential activator of NAMPT at physiological concentrations of the substrates PRPP and Nam.

Our kinetic analyses revealed that millimolar concentration of ATP increases the affinity of NAMPT for Nam and PRPP. In particular, *K_m_* value for PRPP was much higher than its intracellular concentration in the absence of ATP, whereas in the presence of 1 mM ATP, the *K_m_* value decreased to its intracellular concentration. Consistent with the kinetic data, NAMPT did not exhibit significant activity at intracellular concentrations of the substrates without ATP whereas the enzyme was dramatically activated in the presence of millimolar concentrations of ATP. In contrast, at sufficiently high concentrations of the substrates, NAMPT activity was essentially independent of the presence of ATP. These results indicate that millimolar levels of ATP are absolutely required for NAMPT to exhibit significant activity under intracellular milieu, whereas if much higher concentrations of the substrates are available, the nucleotide is not essential for the enzyme activity. Almost the same requirement of ATP was confirmed for human NAPRT. Mutagenesis study identified His-213 as a possible determinant of the activation of NAPRT by ATP. Recently, a similar effect of ATP on the NAMPT reaction has been reported by Burgos and Schramm [26].

We for the first time provided ATP concentrations that produce half-maximal stimulation (*K_a_*) in NAMPT and NAPRT reactions under the physiological condition (Fig. 2). The obtained *K_a_* values for ATP of NAMPT and NAPRT [341±63 µM (*n* = 5) and 395±91 µM (*n* = 3), respectively] were much higher than the reported *K_m_* values for ATP of human NMN/NaMN adenylyltransferase-1, -2, and -3 (59, 89, and 42 µM, respectively) [27] and NAD synthetase (89 µM) [28], the downstream enzymes in the NAD salvage synthesis [1] (Fig. 1). Comparison of these kinetic data revealed that NAMPT and NAPRT are the most sensitive enzymes to changes in cellular ATP levels in the salvage synthesis of NAD. Thus, it is likely that the synthesis of NAD is tightly regulated at the first steps of the pathway by the cellular concentration of ATP, and that based on the millimolar levels of normal cellular ATP this regulation may be significant during substantial depletion of ATP pools, such as that during cellular stress.

It has been hypothesized that visfatin acts as an extracellular NMN-forming enzyme and the resulting NMN is used for intracellular NAD biosynthesis after taken up by cells [9], [11], [14], [15]. However, our study here provides three lines of evidence that do not support this hypothesis. Firstly, the essential requirement of ATP for NAMPT activity does not support NAMPT to catalyze NMN formation in the extracellular milieus. In mouse blood plasma, although the substrate Nam was present at a concentration of 4.6 µM, the other substrate PRPP was not detected and the activator ATP was almost absent (1.3 µM). Taken together with the low affinity of NAMPT for the substrates in the absence of the activator ATP as well as much lower concentrations of the substrates in the blood plasma compared with their intracellular levels, NAMPT should not efficiently catalyze its reaction in the plasma. Secondly, we found that PRPP is rapidly degraded in the plasma. Since the addition of EDTA/EGTA to the plasma inhibited the degradation of PRPP, alkaline phosphatases, with broad substrate specificity towards various phosphated compounds and requirement of metal ions for enzyme activity [16], may at least in part catalyze the degradation. The strong PRPP-degrading enzyme activity in the plasma, as well as the reported short half-life of ATP in the extracellular milieu [16], [29], confirmed in this study, excludes the possibility that extracellular concentrations of PRPP and ATP might rise high enough to support NMN synthesis, such as in the microenvironment of damaged cells. Thirdly, NAMPT added to the plasma did not catalyze NMN formation. The inability to detect NAMPT activity was not due to a small amount of the enzyme added or the inactivation of the enzyme in the plasma, since the enzyme was added to the plasma at a concentration 10 thousand times higher than those in blood plasma [24], [25] and a significant amount of NMN was produced by the addition of PRPP and ATP. These results indicate that NAMPT did not catalyze its reaction in the plasma because of the extremely low concentrations of PRPP and ATP. Thus, NAMPT does not participate in NMN formation under such extracellular milieus. All three lines of evidence provided here indicate that extracellular visfatin can not be considered as an enzyme capable of synthesizing NMN.

We did not detect NMN in the plasma, even with a highly specific and sensitive LC/MS/MS method [18] that can quantify NMN if the mononucleotide is present at a concentration of at least 50 nM. The inability to detect NMN in the plasma is not due to its degradation during the plasma preparation because the nucleotide was stable under the conditions used to prepare plasma. In marked contrast with the present result, previous studies have reported NMN concentrations in blood plasma to be 80–90 µM [9] and culture medium of the muscle cells to be 300 µM [15], using HPLC analysis with UV absorbance detection. The reported high extracellular concentrations of NMN might be arisen from the difficulty of the quantification of NMN by HPLC analysis because of its extremely short elution time [30].

In conclusion, the essential requirement of ATP for NAMPT activity and the inability of NAMPT added to the mouse blood plasma to form NMN indicate that extracellular environments with little or no ATP do not allow extracellular visfatin to catalyze the *in situ* formation of NMN. In addition, extracellular NMN does not serve as a direct precursor of intracellular NAD biosynthesis [31]. Collectively, these observations do not support the concept of “NAMPT-mediated systemic NAD biosynthesis” [9], [14]. The presence of visfatin in circulation may merely be the result of cell death [32], or even if plasma visfatin has physiological functions it may act independent of its NMN biosynthetic activity [33]. Our study would help to clarify physiological significance of extracellular NAMPT.
